# Intermittent Short-Duration Re-oxygenation Attenuates Cardiac Changes in Response to Hypoxia: Histological, Ultrastructural and Oxidant/Antioxidant Parameters

**DOI:** 10.3389/bjbs.2022.10150

**Published:** 2022-03-18

**Authors:** Ayed A. Shati, Mohamed Samir A. Zaki, Youssef A. Alqahtani, Mohamed A. Haidara, Mohammed A. Alshehri, Amal F. Dawood, Refaat A. Eid

**Affiliations:** ^1^ Department of Child Health, College of Medicine, King Khalid University, Abha, Saudi Arabia; ^2^ Department of Anatomy, College of Medicine, King Khalid University, Abha, Saudi Arabia; ^3^ College of Medicine, Zagazig University, Zagazig, Egypt; ^4^ Department of Physiology, Kasr al-Aini Faculty of Medicine, Cairo University, Cairo, Egypt; ^5^ Department of Basic Medical Sciences, College of Medicine, Princess Nourah bint Abdulrahman University, Riyadh, Saudi Arabia; ^6^ Department of Pathology, College of Medicine, King Khalid University, Abha, Saudi Arabia

**Keywords:** intermittent reoxygenation, cardiomyocytes, hypoxia, histology, ultrastructe, TNF-α, IL-6

## Abstract

**Context:** Intermittent short-duration re-oxygenation attenuates cardiac changes in response to hypoxia.

**Objective:** To see if intermittent short-duration re-oxygenation may protect the heart muscle from hypoxia damage.

**Materials and Methods:** Eighteen albino rats were used to carry out the study. Rats divided into: (normoxia); rats exposed to room air as a control, second (hypoxic) group; rats subjected to a pressure of 405 mmHg in a hypobaric chamber to simulate hypoxia at 5,000 m, and third (intermittent short-duration re-oxygenation); rats exposed to room air three times per day. Experiments were all 14 days long.

**Results:** Hypoxia enhanced the oxidative stress biomarker malondialdehyde while lowering the antioxidant superoxide dismutase . The levels of tumour necrosis factor (TNF-α) and interleukin-6 (IL-6) in the myocardium were elevated in hypoxic hearts. The hypoxic rats’ cardiac myofibrils showed disarray of muscle fibres, vacuolation of the sarcoplasm, pyknosis of the nucleus, and expansion of intercellular gaps on histological examination. In addition, cardiomyocytes showed degenerative defects in ventricular myocardial cells on ultrastructural analysis. Myofibril thinning and degenerative mitochondrial changes affected intercalated discs with fascia adherent, desmosomes, and gap junction. Intermittent short-duration re-oxygenation improve cardiac histological, ultrastructural and oxidant/antioxidant parameters changes during hypoxia.

**Conclusion:** Hypoxia showed a substantial impact on myocardial architecture, as well as increased oxidative stress and pro-inflammatory cytokines. Intermittent short-duration re-oxygenation significantly decreases hypoxia-induced cardiac changes.

## Introduction

The oxygenation of tissue is critical to the maintenance and survival of an organ. The effects of chronic hypoxia have been reported in response to high altitude (1) or as a contributing factor in various pathological conditions, such as chronic obstructive pulmonary disease (2), pregnancy complications (3), sleep apnea (4), myocardial infarction (5) and heart failure (6). Excessive oxygen consumption or decreased oxygen supply could lead to insufficient oxygen levels for sustaining normal cellular function, a condition defined as hypoxia (7). Normally, the tissues receive roughly four times more oxygen than they require. To ensure adequate supply, the heart extracts around half of the O_2_ delivered to it (8).

Today, more than 140 million people live at altitudes higher than 3,000 m (9). Hypoxia is caused by a discrepancy in oxygen supply and demand. Tissue hypoxia is produced by a drop in arterial blood oxygen partial pressure and/or an interruption in coronary blood flow (PO_2_ (10). In instances like cardiac arrest, hypoxia and acidosis are important clinical, pathophysiological mechanisms. Both factors have been addressed in a recently published study (11) in resuscitated patients, the dynamic changes in blood oxygen, carbon dioxide, and pH were assessed.

The heart can be subjected to various hypoxic insults, from minor and transitory to long-term and severe. Acute hypoxia has well-documented metabolic effects, including amplified glycolytic flux and temporary lactate acidosis (12, 13). Hypoxia that lasts for a long time or is severe requires cardiac metabolism to reprogramming; the heart decreases oxygen-consuming activities and increases glycolysis to maximize ATP generation when the oxygen level is low (14–16).

The imbalance between the antioxidant defence mechanisms and reactive oxygen species (ROS) production is known as oxidative stress. The reactive oxygen species (ROS) production has played a key role in myocardial damage produced by the heart’s ischemia-reperfusion (17–19). The cardioprotective impact of ROS scavengers and agents capable of generating antioxidants such as superoxide dismutase (SOD) and augmenting antioxidants have provided indirect evidence supporting this hypothesis (20). The present work aims to evaluate the effect of intermittent short term re-oxygenation on hypoxia-induced cardiac changes.

## Materials and Methods

### Animals

Twelve weeks old, male Sprague–Dawley rats (350–400 gm), were purchased from the Experimental Research Laboratory of King Khalid University. The temperature was kept at 25°C. Animals were maintained on 12 h–12 h light-dark cycles. Rats were fed standard laboratory chow (Laboratory Diet 5001; PMI Nutrition International Inc., Brentwood, MO, United States) and water ad libitum. The project was approved by the ethical committee of King Khalid University, College of Medicine (Abha, KSA).

### Experimental Design

Eighteen rats were randomly categorized into three groups (*n* = 6): normoxia, hypoxia, and intermittent re-oxygenation as follow: 1) Control group (normoxia): rats were exposed to 400 m (laboratory altitude, gas composition: 21% O2-79% N2) for 24 h each day.; 2) Hypoxia group: rats were exposed to a hypobaric chamber with a pressure of 405 mmHg to simulate hypoxia at about 5,000 m for 24 h each day 3) Intermittent 3-h re-oxygenation group: rats were treated similarly as group Hypoxic group, with 42% O2-58% N2 intermittently for 3 h each day.

We set the re-oxygenation level to 0 m, the duration of each session to 1 h, and the frequency to two, three, and six times each day after a 2-week treatment (21). The authors added that wash off CO_2_, H_2_O, and NH_3_, the hypobaric chamber was continually flooded with room air or 42 per cent O_2_-58 per cent N_2_. Sodium lime and discoloured silica gel were utilised to absorb extra water and carbon dioxide in each chamber. Every 2 days, each chamber was opened for 20–25 min to clean the cages and restock food and water.

### Measurement of MDA and SOD, Indicators of Oxidative Stress

Cardiac tissues were homogenized in ice-cold saline and then centrifuged for 15 min at 18,000 g (148C). Spectrophotometric measurement of MDA (indicative of lipid peroxidation). was measured using the thiobarbituric acid substrate assay (TBARS Assay Kit, Item No. 10009055, Cayman Chemical Company, Ann Arbor) as an indicator (22). SOD (an antioxidant measured using SOD Kit (Item No. 706002, Cayman Chemical Company, Ann Arbor (23).

### Determination of the Levels of IL-6 and TNF-α

Cardiac tissue homogenate was used to determine the levels of TNF-α (ELISA kit BIOTANG INC., Cat. No. R6365, MA, United States) (24) and IL-6 (ELISA kit BIOTANG INC., Cat. No. RB1829, MA, United States) according to the manufacturer’s directions.

### Haematoxylin–Eosin Staining of the Heart Cross-Sections

The heart was perfused by normal saline followed by 10% neutral buffered formalin to ensure optimal fixation before being processed into wax. Three millimetres of specimens from cardiac tissues were cut. Specimens were cleaned overnight with tap water followed by the dehydrating step by passing the specimens through graded series of ethanol from (50% up to 100%), then incubated in a mix of 100% ethanol (50:50) and xylene for 30 min. After that, they were transferred into a paraffin and xylene mixture for 30 min. After that, the specimen was thoroughly infiltrated with wax, then formed into a “block” which can be clamped into a microtome for section cutting. This step was carried out using an “embedding centre” where a mold was filled with molten wax and the specimen placed into it. The specimen is very carefully orientated in the mold. A cassette is placed on top of the mold, topped up with more wax, and the whole thing was placed on a cold plate to solidify. When this was completed, the block with its attached cassette was removed from the mold and were ready for microtomy. 5 μm sections were taken by Microtomes (Epredia HM 325, Unites States), designed to be precise and stable, helping to yield superior sectioning results. Serial sections were prepared and stained for sequential analysis. The sections were then dewaxed under 60°C and dipped in xylene for 1 hour and rehydrated through a series of graded ethanol (100%, down to 60%) for 5–10 min, each every 2 min, then stained with hematoxylin [(Mayer’s, Modified) (ab220365) Abcam, United Kingdom)] for 5–10 min then washed in water followed by soaking in warm water till they become bright violet, then immersed in Eosin solution (Eosin Y Solution (Aqueous) (ab246825) Abcam, United Kingdom) for 3–5 min. Each slide was then soaked in 85% and 100% alcohol for 15 min each. The slides were then immersed twice in xylene and mounted using an Entellan embedding agent and covered with coverslips before viewing. Photographs were taken using the Olympus light microscope (OYS_CH20i) provided by a digital camera connected to a computer (25).

### Transmission Electron Microscopy (TEM)

Heart specimens were immediately preserved and fixed in a 2.5 per cent glutaraldehyde solution (Glutaraldehyde 50% CAS 111-30-8) in 0.1 M sodium cacodylate buffer, pH 7.2, trimmed and sliced into one cubic millimetre pieces, and stored at 4°C for 2 hours. They were post-fixed in a sodium cacodylate buffer (Shanghai Sunwise Chemical Co., Lt China) containing 1% osmium tetroxide, then dehydrated in an ascending series of ethyl alcohol before being fixed in Spur’s resin. Then examined under the Electron microscope (JEM-1011, Jeol Co., Japan) operating at 80 Kv (26).

### Statistical Analysis

Data were processed and analyzed using the SPSS version 10.0 (SPSS, Inc., Chicago, IL, United States) and was presented as mean ± standard deviation (SD). One-way ANOVA was used followed by Tukey’s post hoc test. Pearson correlation statistical analysis was carried out to detect a probable significant difference between the two parameters. Results were considered significant if *p* ≤ 0.05.

## Results

### Biochemical Markers of Pro-Inflammatory Cytokines and Oxidative Stress

**FIGURE 1:**
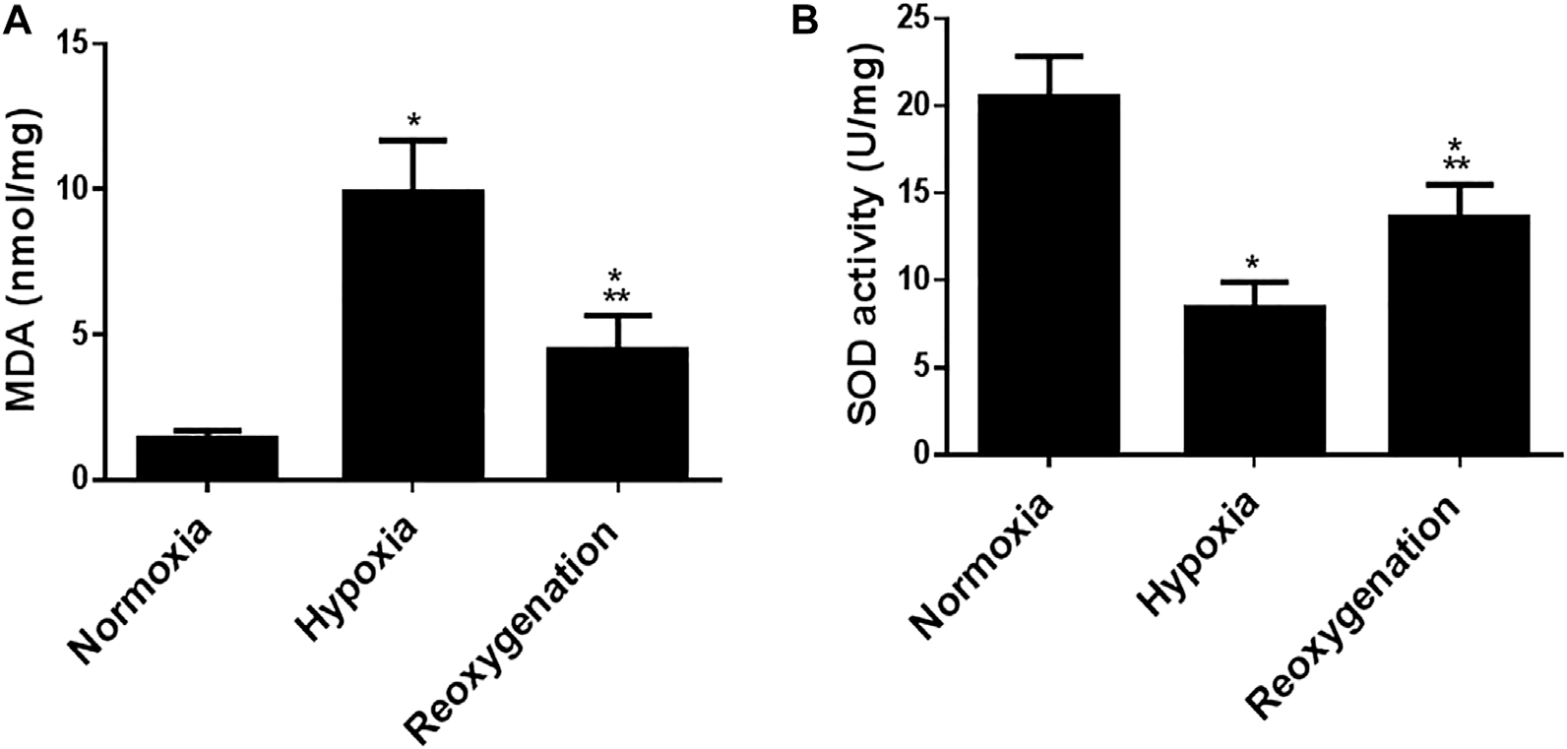
The effect of intermittent re-oxygenation on oxidative stress biomarkers in rats. Heart homogenates levels of MDA **(A)** and SOD **(B)** were assessed in different groups of rats at the end of the experiment; Normoxia group, hypoxia group, and re-oxygenation group. Results were represented as mean (±SD); *n* = 6 for each group. Experiments were performed in triplicate. *p < 0.05 versus normoxia, **p < 0.05 versus hypoxia.

**FIGURE 2:**
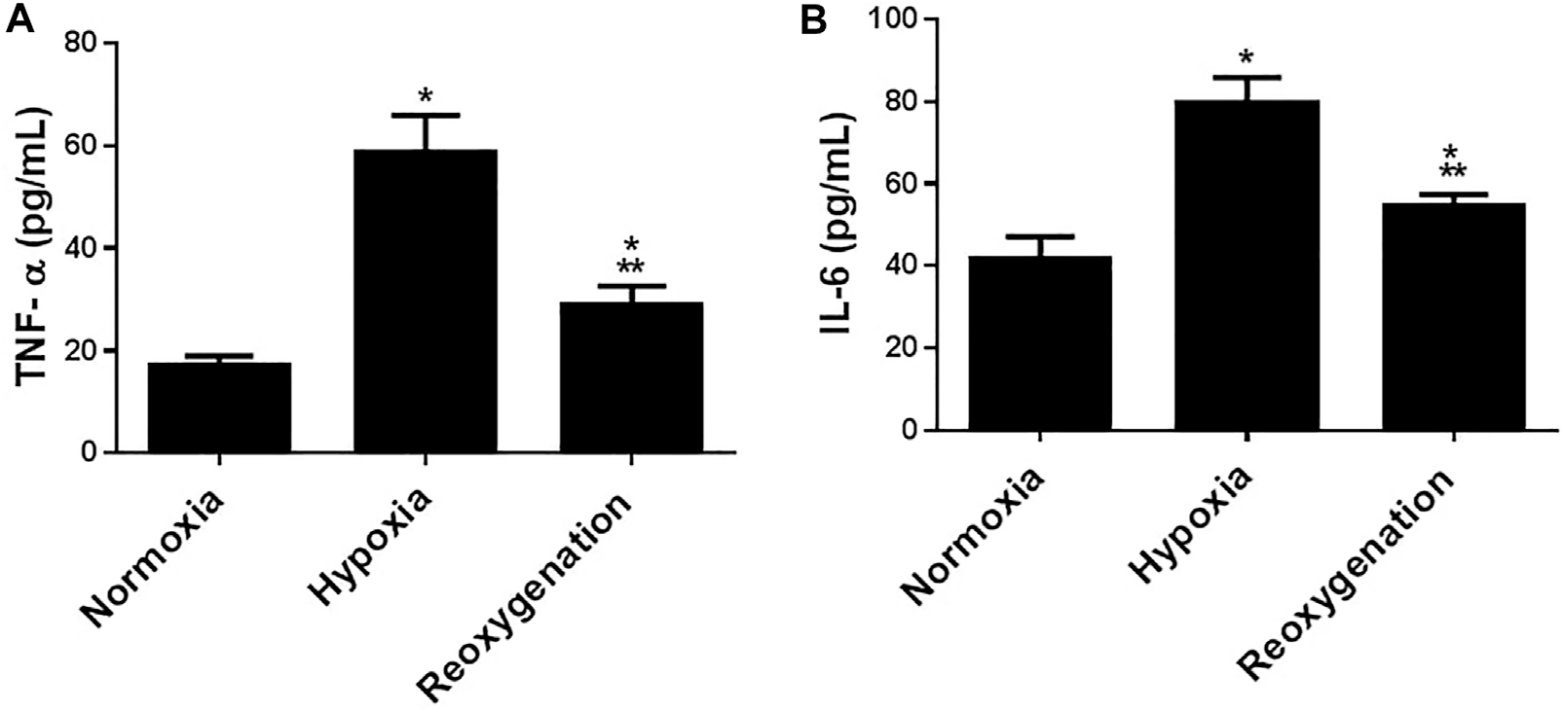
The effect of intermittent re-oxygenation on proinflammatory biomarkers in rats. TNF-a **(A)** and IL-6 **(B)** levels in the heart tissue were assessed at the end of the experiment in the different groups of rats; Normoxia group, hypoxia group, and re-oxygenation group. Results represent the mean (±SD); *n* = 6 for each group. Experiments were performed in triplicate. *p < 0.05 versus normoxia, **p < 0.05 versus hypoxia.

To explore the effect of intermittent short-duration re-oxygenation on proinflammatory cytokines (TNF-α and IL-6) and oxidative stress biomarkers (MDA and SOD) in cardiac tissues were measured. Figures 1A,B showed that hypoxia significantly (*p* < 0.05) increased proinflammatory cytokines, which were significantly decreased by intermittent short-duration re-oxygenation compared to the hypoxia group, but it did not bring it back to the control level. Hypoxia significantly (*p* < 0.05) amplified the oxidative stress biomarkers MDA (Figure 2A) and decreased the antioxidant SOD (Figure 2B). Intermittent short-duration re-oxygenation caused decreased MDA and increased SOD significantly in comparison to the hypoxia group but did not return to the control level.

### Histological Study

**FIGURE 3:**
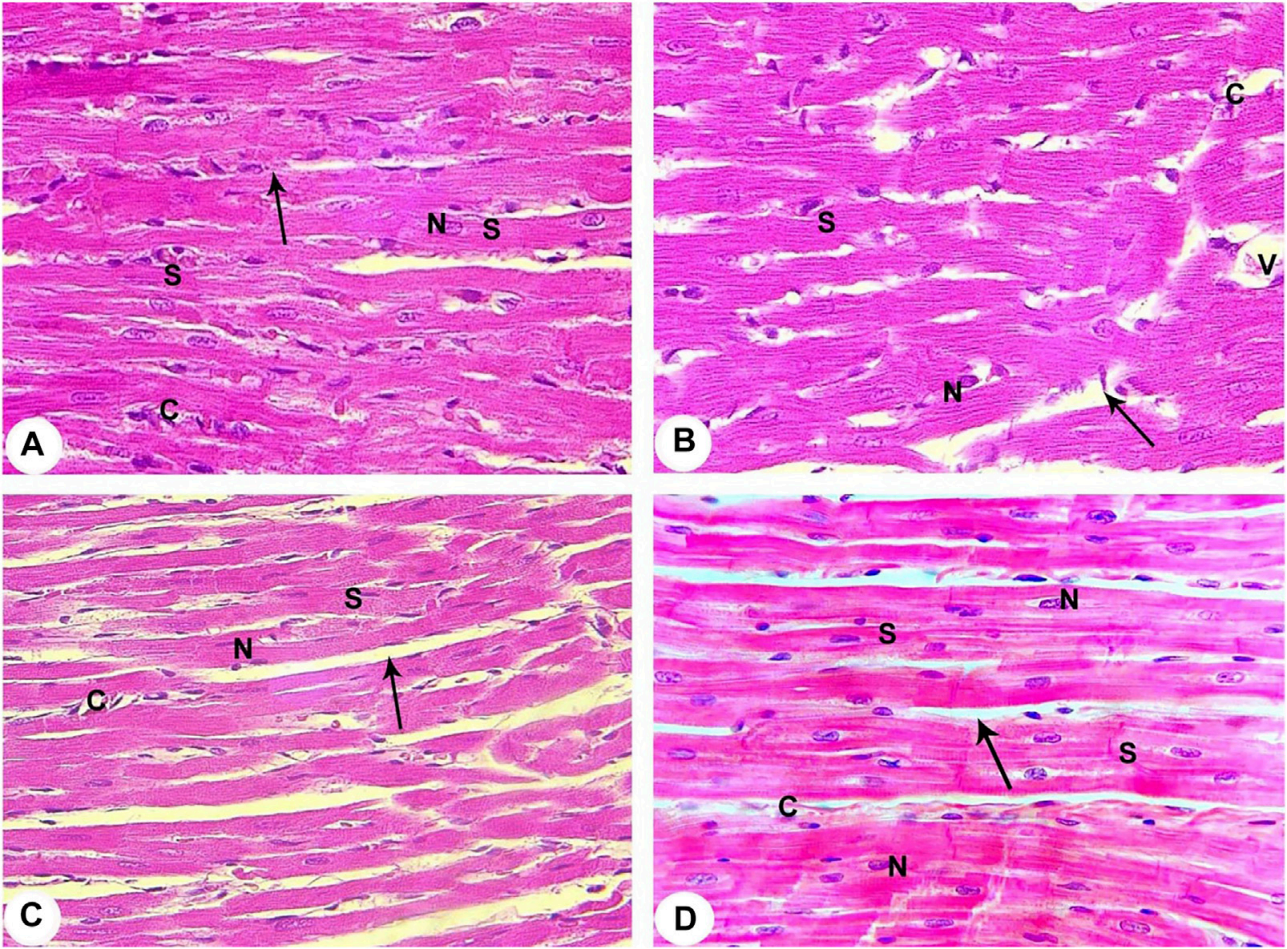
Histopathological evaluation from all groups of rats (400X). **(A)** Contol (Normoxia) group: The rat’s cardiac muscle structure shows centrally located nuclei **(N)**, branching cytoplasmic network with striations **(S)**, and delicate connective tissue between the muscle fibres **(arrow)** with few capillaries **(C)**. **(B)** Hypoxia group: Rats in the hypoxia showed more apparent alterations, where most of the cardiac muscle fibre striations **(S)** have disorganization and fragmentation, increment of nuclear peripheralization and pyknosis **(N)** together with sarcoplasmic vacuolation. Increased interstitial space **(arrow)** with congestion **(V)** and dilation of the blood capillaries **(C)** are displayed. **(C)** Hypoxia group: The rats of this group showed disruption of cardiac myocyte, degeneration of muscle fibres **(S)**, pyknotic nuclei **(N)**, congestion and dilation of the myocardial capillaries **(C)** are displayed. A marked increase of the interstitial space **(arrow)** is also noticed. **(D)** Intermittent short-duration reoxygenated group: The rat’s cardiac muscle after recovery showed the approximately normal histological appearance of cardiac myocyte striations **(S)**, minimal interstitial space **(arrow)**, decreased congestion of blood capillaries **(C).** Some pyknotic nuclei **(N)** are still seen.

The control group showed that cardiac muscle fibres striated and arranged in a linear array that anatomizes and branches in a certain pattern (sheet-like). They contain acidophilic cytoplasm with centrally located oval nuclei. A delicate layer of connective tissue separates the cardiac muscle fibres with well-evidenced myocardial blood capillaries (Figure 3A).

The hypoxic group revealed disruption, degeneration, and disorganization of the cardiomyocytes. These cardiomyocytes revealed varying degrees of enlargement. Most of the myocytes contain pale acidophilic sarcoplasm with peripherally situated nuclei. The nuclei were pyknotic and enclosed by perinuclear cytoplasmic vacuolation. There is a widening of the inter-fibre where they enclose congested dilated blood vessels. Moreover, perivascular and focal mononuclear cellular infiltration was worth noting. Furthermore, areas of hemorrhage and interstitial edema were observed (Figures 3B,C).

The intermittent re-oxygenation group ameliorated myocardial injury demonstrated by decreased myonecrosis, edema and red blood cells extravasations associated with minimum inflammation. Many well-organized muscles fibres with central vesicular nuclei were observed, while some fields showed few muscles fibres with peripheral dark nuclei and slightly rarified cytoplasm. There were also a few dilated congested blood vessels present between muscle fibres and a few areas of blood extravasation (Figure 3D).

### Transmission Electron Microscopy (TEM) Analysis

**FIGURE 4:**
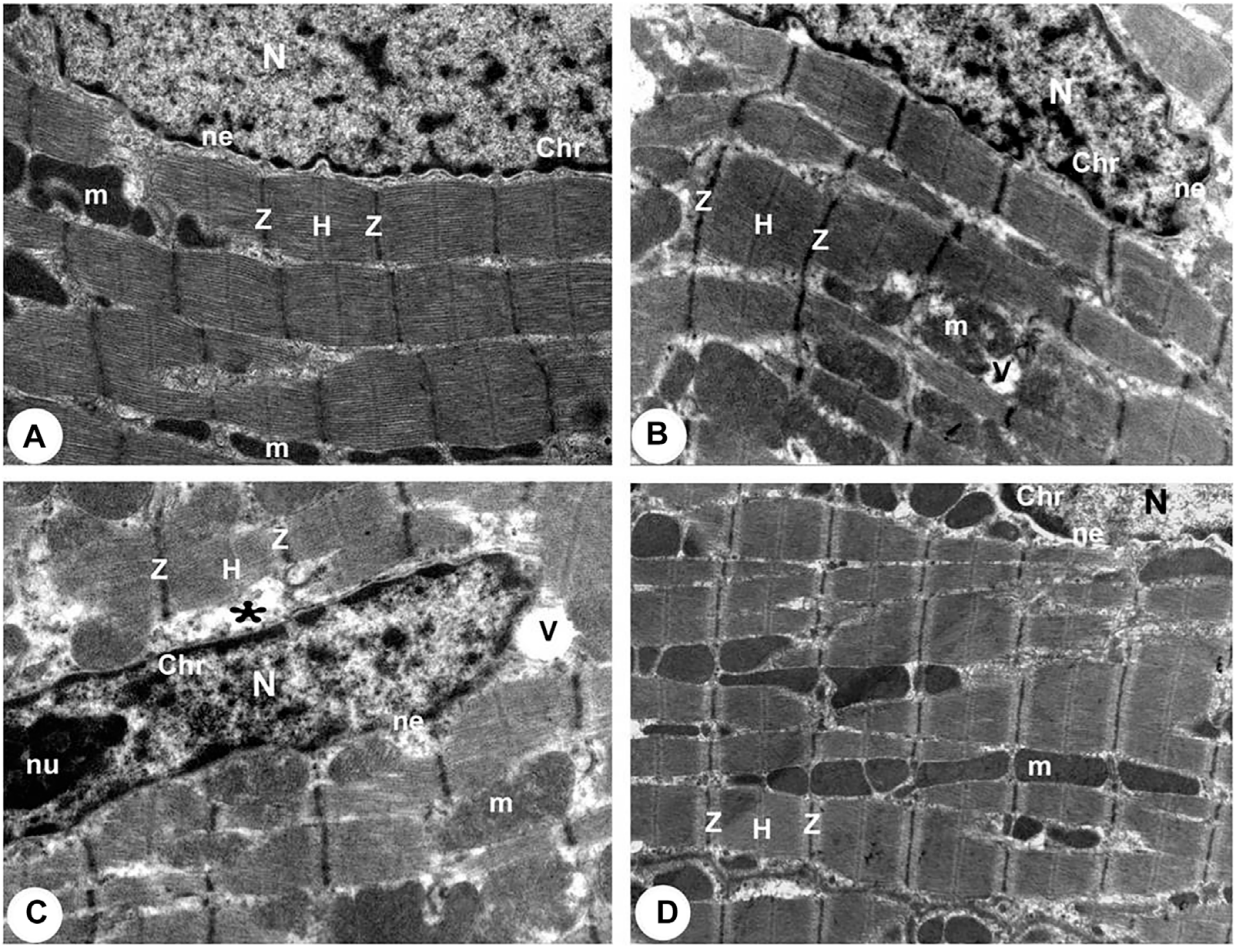
Transmission electron micrographs obtained from all groups (100,00X). **(A)** Normoxia group rats showed a normal myocardial striations architecture with well preserved mitochondrial (m) integrity. Dense cytoplasm filled with healthy myofibrils and its striated pattern with clear bands (Z and H), intact nucleus with a normal distribution of chromatin (Chr), and nuclear envelope (ne). **(B)** Hypoxia group rats showing slightly disrupted myofibrils with minimal disarrangement of muscle bands **(Z and H)** and swollen mitochondria **(m)** with disoriented and dissolution cristae, flocculent density deposition and breaks in the outer mitochondrial membrane. Disassembly of the nuclear envelope **(ne)**, clumped chromatin **(Chr)** randomly dispersed in the nuclei **(N)**, and a few vacuoles **(V)** are also observed. **(C)** Hypoxia group rats showed marked myofibrils disruption with disarrangement of muscle bands (Z and **H**) and swollen mitochondria (m) with disoriented and dissolute cristae, flocculent density deposition, and breaks in the outer mitochondrial membrane. Disassembly of the nuclear envelope **(ne)**, clumped heterochromatin randomly dispersed in the disrupted nuclei **(N)**, few vacuoles **(V)** and marked spaces interstitially (*) are also observed. **(D)** Intermittent short-duration reoxygenated group rats: showing normal architecture with well-preserved integrity and striated pattern of clear bands **(Z and H)**, nucleus **(N)** with a normal distribution of chromatin **(Chr)** and nuclear envelope **(ne)** and mitochondria **(m)**.

**FIGURE 5:**
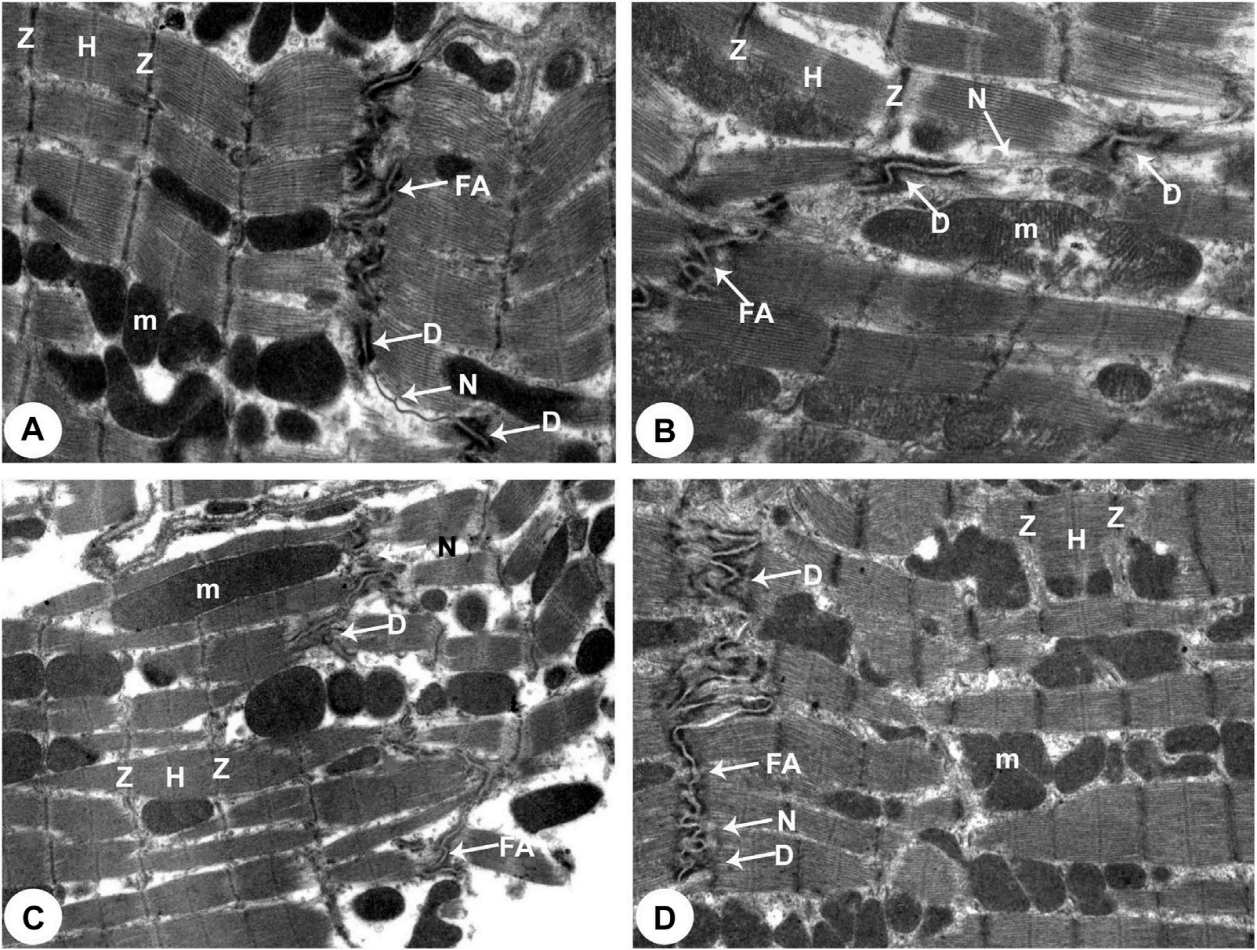
Transmission electron micrographs of the rats attained from all groups of rats (Intercalated disc with fascia adherent) (100,00X). **(A)** Normoxia group rats showed normal cardiocyte structure with dense cytoplasm comprising healthy myofibrils and its striated pattern with clear bands (Z and H) and intact mitochondria (m). Intercalated disc with characteristic fascia adherent (FA), desmosomes (D) and gap junction (N) are also seen. **(B)** Hypoxia group rats showed disrupted cytoplasm of myofibrils with the fragmentation of muscle bands (Z and H) and pleomorphic mitochondria (m). Damaged intercalated discs with fascia adherent (FA), desmosomes (D) and gap junction (N) are also seen. **(C)** Hypoxia group rats showed marked degeneration of the cytoplasm of myofibrils with the fragmentation of muscle bands (Z and H) and pleomorphic mitochondria **(m)**. Damaged intercalated discs with fascia adherent **(FA)**, desmosomes **(D)** and gap junction **(N)** are also seen. **(D)** Intermittent short-duration reoxygenated group rats: showing normal myofibrils with a striated pattern of clear bands **(Z and H)**, mitochondria **(m)** and intercalated discs with fascia adherent **(FA)**, desmosomes **(D)** and gap junction **(N)** are also seen.

**FIGURE 6:**
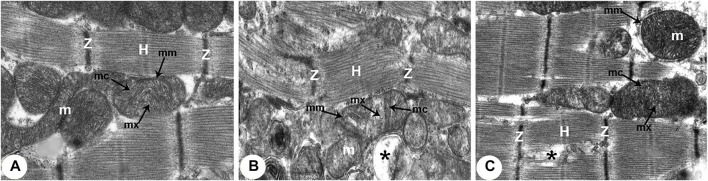
Transmission electron micrographs of the rats obtained from all groups (300,00X). **(A)** Normoxia group rats showing normal cardiocyte structure with dense cytoplasm filled with normal myofibrils with striated pattern and clear bands (Z and H) and evenly distributed mitochondria (m) as chain-like structures closely related to the length of sarcomeres. Mitochondria are usually oval, containing outer and inner membrane (mm) transversally oriented cristae (mc) within a moderately electron-dense mitochondrial matrix (mx). **(B)** Hypoxia group rats showing disruption of contractile elements with multifocal lysis of myofibrils (Z and H) and severe mitochondrial (m) degenerative alterations compared with the normal ultrastructure in control rats. The mitochondrial inner membrane (mm) is destroyed into the distended matrix (mx) and the cristae (mc) fragmentation or central effacement (*). **(C)** Intermittent short-duration reoxygenated group rats: showing almost normal cardiac muscle fibres with a striated pattern of clear bands (Z and H). Mitochondria (m) retained their intact cristae (mc) and dense matrix (mx), while few appeared swollen (*) with an irregular shape, size, and partially devastated cristae.

According to TEM photomicrographs, the ventricular myocytes in the control group were branched, striated, and connected at the intercalated discs (ICDs). Sarcomeres are arranged in a well-defined and visible pattern. Myofibrils were grouped in a regular pattern within the sarcomeres and extend between the bands (Z and H) and the intercalated disc with fascia adherent, desmosomes, and gap junction. Ovalwell organized, closely attached mitochondria were distributed between the myofibrils. The ventricular myocytes’ nuclei were euchromatic, with homogeneous, loose, and finely granular chromatin. Furthermore, a dense thin layer of chromatin was already identified near the nuclear membrane’s inner surface. ICDswere preserved. The finger-like folds of ICDs, called interdigitations, were repeated at regular intervals. Within the interdigitations, there were fasciae adherents, spot desmosomes and, more to the side, gap junctions (nexus). Also, electron micrographs presented evenly distributed mitochondria as chain-like structures closely linked to the length of sarcomeres. They assembled clusters along myofibrils in all sections of the cardiomyocyte (subsarcolemmal, intermyofibrillar, and perinuclear) in control rats. Mitochondria are oval, containing transversally oriented cristae inside a moderately electron-dense mitochondrial matrix (Figures 4A, 5A, 6A).

There were widespread degenerative abnormalities in some ventricular myocardium cells, as well as some myofibril thinning and rupture, as indicated by TEM photomicrographs of the hypoxic groups. Furthermore, this group had more degenerative mitochondrial alterations. The ICDs with fascia adherent, desmosomes, and gap junction were deteriorated, disorganized, and disrupted throughout the tissue mainly at the site of the adherent junction, with relatively preserved desmosomal intercellular connections (Figures 4B, 5B). The mitochondrial inner membrane changed into the swollen matrix and the cristae fragmentation in most the muscle cells. Disassembly of the nuclear envelope clumped heterochromatin randomly dispersed in the disrupted nuclei, few vacuoles, marked spaces interstitially (Figures 5B,C, 6B).

The intermittent re-oxygenation group showed regeneration of cardiac muscle fibers in comparison to the hypoxic group. The nuclei of the myocytes seemed oval euchromatic. There are regularly arranged myofibrils with regular striation patterns and average size and. ICDs ultrastructure was preserved and appeared to be intact interdigitation of ICDs with recovered fascia adherent. Most of the mitochondria retained their intact cristae and dense matrix, while only a few appeared enlarged with unequal size and shape, size associated with partly destroyed cristae (Figures 4D, 5D, 6D).

## Discussion

Using histological, ultrastructural, oxidant/antioxidant characteristics, we investigated the protective impact of intermittent short-duration re-oxygenation on heart damage generated by hypoxia in a rat model.

MDA is a highly reactive aldehyde that originates from polyunsaturated fatty acid lipid peroxidation. This aldehyde’s synthesis is used to diagnose oxidative stress (27). MDA levels increased in response to hypoxia-induced oxidative stress in the current investigation. MDA, a lipid peroxidation breakdown product, is a marker of cellular injury severity to the heart caused by hypoxia and can be associated with the structure of the altered membrane (28).

Our study showed low MDA levels in the reoxygenated group can be caused by enhanced actions of the antioxidant defence in the cardiac muscles.

The enzymatic antioxidant defence, which includes SOD, is one of the mechanisms that defend the host cells from excess free radicals. SOD is an important enzyme that appears to be the first line of defence against ROS. Our results showed an increased level of cardiac tissue SOD after intermittent re-oxygenation, indicating that re-oxygenation may have antioxidant and free radical scavenging properties (29, 30).

The cytokines IL-6 and TNF-α were increased in cardiac homogenate in hypoxic hearts compared to normoxic hearts (31). According to our findings, the cytokines IL-6 and TNF-α may have a significant role in cardiac tissue derangement observed in our study. Infusion of TNF-α into rats at comparable levels to those found in patients with end-stage heart failure leads to cardiac myocyte shortening, left ventricular dilation and depression of left ventricular function (32). Consistent with our study, cardiac myocytes have been shown to produce significant amounts of TNF-α (33). As a result, Il-6 and TNF-α generated by cardiac myocytes likely play a key role in heart tissue malfunction (34).

Intermittent hypoxia and the consequent re-oxygenation result in ROS production, resulting in systemic oxidative stress (35). ROS can react with nucleic acids, lipids, and proteins, leading to DNA alterations, inflammation and cellular damage (36). Moreover, intermittent hypoxemia stimulates the release of pro-inflammatory factors and promotes metabolic dysregulation (37).

Histological examination of the heart specimens of the hypoxic rats showed pathological changes in all of the specimens. The most significant changes were remarkable widening of the inter-fibre spaces with disarrangement of the cardiac architecture. Hypoxia caused broadening of the interstitial tissue in cardiac muscles (38) and could be due to increased connective tissue elements, particularly collagen fibres, and was associated with the level of muscle injury. Hypoxia also caused significant cardiomyocytes edema and degeneration of myofilaments (39). The reoxygenated group demonstrated protection from myocardial injury caused by hypoxia, also shown by decreased myonecrosis and edema, and minimal inflammation. Well-organized muscle fibres with central vesicular nuclei are observed, while some fields displayed few muscle fibres with peripheral with slightly rarified cytoplasm.

TEM examination showed myofibrillar disruption in the cardiomyocytes of the hypoxic rats’. In addition, certain myocytes revealed loss of striation in the form of indistinct Z-lines, A and H zones were nearly invisible, and the intercalated discs’ unique shape was lost. Activation of calcium stimulated proteinase in necrotic muscle may be responsible for myofibril disintegration (40). Mitochondria are essential for various physiological activities, including the balance of pro-and antioxidant mechanisms involved in the development of many cardiovascular illnesses (41). Furthermore, mitochondria are in charge of generating energy through oxygen and nutrient management and controlling cellular metabolism and apoptosis (42). Kotamraju et al. (43) attributed mitochondrial alterations to an increase in the cytoplasmic portion of cytochrome C, and decreased translocation of mitochondrial cytochrome C. Nitric oxide (NO)-related cardiomyocyte damage was further demonstrated by increased mitochondrial nitrotyrosine production. However, NO has been linked to cytotoxicity since it has been shown to disrupt mitochondrial respiratory chain enzymes and cause mitochondrial-induced apoptosis. Changes in mitochondrial size and the number of cristae per mitochondrion are linked to changes in mitochondrial enzyme activity (44). Most of the mitochondria reserved their dense matrix and intact cristae whereas a few were engorged with an irregular shape, size and partly damaged cristae in the reoxygenated treated group.

The cardiomyocytes nuclei in the hypoxia groups showed varying degrees of chromatin density reduction and karyolysis and in the current investigation. Several nuclear membrane invaginations and diverse chromatin abridgement were also observed. The cardiac myocytes and their mitochondrial contents were disrupted, resulting in perinuclear edema. An interpretation is comparable with (45) who documented that severe myocardial ischemia is associated with chromatin clumping, shrivelled nuclei which led to cardiomyocyte necrosis in dogs. Capillary injury in the heart’s vasculature, on the other hand, could explain the myofilament loss and myofibrillar destruction seen in our findings. Over-expression of serum deprivation protein response and transcript release factor, unlike polymerase I, may have generated caveolae deformation and severe tubulation of the endothelial cell membrane in this situation, as previously documented (46).

In this current research, intermittent short-duration re-oxygenation effectively reduces hypoxia-induced cardiac architectural changes. Cardiac tissues show an improved cardiac muscle fibres appearance with a striated arrangement of Z and H bands and reduced structural remodeling of cardiac muscle cells that occurred under hypoxia. Data showed that RNA sequencing identified the specific genes and molecular pathways that mediate the protective effects (21).

Preclinical studies of a mice model of heart failure (47) showed an improved cardiac function following exposure to intermittent hypoxia. While current preclinical and clinical results suggest that intermittent hypoxia (Hypoxia plus intermittent re-oxygenation) may be a non-pharmacological therapeutic and innovative choice in cardiovascular diseases, more clinical studies are needed in patients with various cardiac conditions to approve the possible use of intermittent hypoxia as a therapeutic tool.

### Conclusion

Based on our findings, intermittent short-duration re-oxygenation reduces the cardiac architectural changes caused by hypoxia due to decreased oxidative stress and pro-inflammatory cytokines.

## Data Availability

The raw data supporting the conclusions of this article will be made available by the authors, without undue reservation.
